# Supplementary material for: Emergency Foot-and-Mouth Disease Vaccines A Malaysia 97 and A22 Iraq 64 Offer Good Protection against Heterologous Challenge with A Variant Serotype A ASIA/G-IX/SEA-97 Lineage Virus
Source: Vaccines (Basel). 2020 Feb 10;8(1):80. doi: 10.3390/vaccines8010080 (PMC7157217; doi:10.3390/vaccines8010080)
Supplement: Supplementary file 1 [file vaccines-08-00080-s001.pdf]

Supplementary Table S1a: Viraemic Phase: qPCR results indicating presence of FMDV RNA (the Cp values) in blood, oral swabs, nasal swabs and probang samples from 0 to 35 days post challenge. Animals were vaccinated with two different vaccines and challenged at 7- or 21-days post vaccination with a heterologous virus along with UVC group.

Groups	Calf ID	0 dpc				1 dpc			2 dpc			3 dpc			4 dpc			5 dpc			6 dpc			7 dpc			
		B	OS	NS	P	B	OS	NS	B	OS	NS	B	OS	NS	B	OS	NS	B	OS	NS	B	OS	NS	B	OS	NS	P
	8092	45.00	45.00	45.00	45.00	29.71	13.70	45.00	45.00	14.39	29.50	45.00	29.15	15.74	45.00	21.19	30.05	45.00	19.50	31.69	45.00	32.35	45.00	45.00	32.37	32.41	30.06
	8093	45.00	45.00	45.00	45.00	45.00	28.59	30.16	45.00	25.78	28.80	45.00	28.73	15.70	45.00	19.27	31.18	45.00	19.98	32.18	45.00	18.77	45.00	45.00	18.79	32.81	22.57
**A22 IRQ-21**	8094	45.00	45.00	45.00	45.00	32.17	7.40	30.36	45.00	13.71	28.60	29.89	24.04	16.63	31.65	18.75	29.12	45.00	20.11	30.51	45.00	25.31	32.10	45.00	32.60	45.00	30.59
	8095	45.00	45.00	45.00	45.00	30.45	12.04	45.00	45.00	14.77	29.70	45.00	27.17	16.62	45.00	22.51	31.74	45.00	21.23	32.13	45.00	21.99	32.58	45.00	18.67	45.00	22.12
	8096	45.00	45.00	45.00	45.00	30.52	18.13	45.00	45.00	17.62	30.30	45.00	29.56	21.74	45.00	14.07	45.00	45.00	14.90	32.31	45.00	20.83	31.92	45.00	22.90	45.00	25.78
	8097	45.00	45.00	45.00	45.00	45.00	6.73	45.00	45.00	12.88	28.50	45.00	45.00	17.95	45.00	19.21	31.84	45.00	19.29	45.00	45.00	25.14	32.72	45.00	31.77	45.00	30.57
	8098	45.00	45.00	45.00	45.00	45.00	9.99	45.00	45.00	10.81	30.20	45.00	29.82	18.54	45.00	20.62	31.48	45.00	16.42	32.03	45.00	17.68	45.00	45.00	16.85	31.81	19.74
**A MAY97-21**	8099	45.00	45.00	45.00	45.00	45.00	23.11	45.00	45.00	13.66	29.90	45.00	29.68	18.47	45.00	22.59	31.54	45.00	29.64	31.85	45.00	31.28	45.00	45.00	32.69	32.30	30.47
	8100	45.00	45.00	45.00	45.00	45.00	29.92	45.00	45.00	16.82	45.00	45.00	29.86	19.44	45.00	22.96	31.65	45.00	28.99	31.94	45.00	31.54	45.00	45.00	31.76	45.00	30.41
	8101	45.00	45.00	45.00	45.00	26.94	7.89	45.00	45.00	14.89	30.20	45.00	28.84	16.25	45.00	21.19	29.84	45.00	27.07	31.89	45.00	25.02	32.88	45.00	21.28	45.00	21.07
	8102	45.00	45.00	45.00	45.00	45.00	8.56	45.00	45.00	20.02	45.00	45.00	28.48	20.65	45.00	19.03	31.78	45.00	13.92	31.85	45.00	17.57	32.07	45.00	20.16	30.92	22.71
	8103	45.00	45.00	45.00	45.00	45.00	12.61	45.00	45.00	13.87	45.00	45.00	28.53	20.65	45.00	19.95	31.78	45.00	19.81	31.85	45.00	18.49	32.07	45.00	18.62	30.92	22.26
**A22 IRQ-7**	8104	45.00	45.00	45.00	45.00	29.87	7.88	45.00	45.00	13.46	28.70	45.00	28.09	18.77	45.00	21.00	30.59	45.00	26.89	30.03	45.00	28.14	30.15	45.00	31.12	31.18	30.75
	8105	45.00	45.00	45.00	45.00	45.00	12.16	45.00	45.00	17.40	29.30	45.00	29.59	19.48	45.00	16.04	32.07	45.00	17.82	45.00	45.00	17.22	45.00	45.00	25.91	45.00	26.50
	8106	45.00	45.00	45.00	45.00	29.83	11.64	45.00	45.00	12.33	29.80	45.00	45.00	19.58	45.00	18.89	31.16	45.00	16.93	32.00	45.00	17.90	32.21	45.00	19.36	32.02	19.09
	8107	45.00	45.00	45.00	45.00	45.00	17.05	30.45	45.00	15.44	29.70	45.00	45.00	21.38	45.00	24.59	31.63	45.00	22.14	45.00	45.00	26.56	32.14	45.00	27.76	45.00	27.74
	8108	45.00	45.00	45.00	45.00	45.00	16.81	45.00	45.00	13.06	29.00	45.00	29.68	20.62	45.00	18.79	30.50	45.00	20.07	45.00	45.00	23.20	45.00	45.00	25.34	32.61	27.28
**A MAY97-7**	8109	45.00	45.00	45.00	45.00	28.45	7.62	29.58	45.00	16.03	23.00	29.60	29.23	20.69	45.00	23.67	28.53	45.00	18.44	45.00	45.00	19.25	32.76	45.00	21.01	32.04	23.77
	8110	45.00	45.00	45.00	45.00	30.99	12.59	45.00	45.00	13.00	30.50	45.00	30.53	19.56	45.00	19.08	31.80	45.00	18.69	32.17	45.00	18.15	32.54	45.00	26.02	32.70	23.50
	8111	45.00	45.00	45.00	45.00	25.46	18.85	20.10	28.56	14.50	20.20	24.44	17.03	17.92	32.07	19.28	25.31	45.00	24.55	21.27	45.00	28.47	29.90	45.00	23.43	28.45	25.08
	8112	45.00	45.00	45.00	45.00	29.86	27.79	20.09	28.60	13.92	20.70	19.64	21.62	15.86	29.64	18.85	27.11	32.03	21.97	29.70	45.00	25.41	29.84	45.00	27.89	31.51	27.45
**UVC**	8113	45.00	45.00	45.00	45.00	30.31	9.73	22.71	28.15	14.39	27.90	20.61	27.46	19.27	29.79	20.72	29.91	32.65	17.44	31.69	45.00	17.38	31.85	45.00	21.46	45.00	21.11
	8114	45.00	45.00	45.00	45.00	29.21	5.00	21.97	28.01	10.54	23.40	19.88	20.69	15.19	30.44	21.81	20.79	32.31	45.00	28.49	45.00	20.91	25.01	45.00	17.10	29.97	15.67

dpc – day post challenge; S – Serum, OS – Oral Swab, NS – Nasal Swab, P – Probang sample,
Cells with grey shade are positive for FMDV genome; Numerical values indicate the Cq values of qPCR; X – No sampling done on that day and * - Cattle that were not protected upon challenge

Supplementary Table S1b: Post-viraemic Phase: qPCR results indicating presence of FMDV RNA (the Cp values) in blood, oral swabs, nasal swabs and probang samples from 0 to 35 days post challenge. Animals were vaccinated with two different vaccines and challenged at 7- or 21-days post vaccination with a heterologous virus along with UVC group.

Groups	Calf ID	10 dpc				14 dpc				21 dpc				28 dpc				30 dpc				30 dpc			
		B	OS	NS	P	B	OS	NS	P	B	OS	NS	P	B	OS	NS	P	B	OS	NS	P	B	OS	NS	P
	8092	45.00	45.00	45.00	31.10	45.00	45.00	45.00	29.43	45.00	45.00	45.00	31.06	45.00	45.00	45.00	29.40	X	X	X	32.05	45.00	45.00	45.00	29.32
	8093	45.00	31.65	45.00	29.95	45.00	45.00	45.00	45.00	45.00	45.00	45.00	31.07	45.00	45.00	45.00	29.46	X	X	X	30.80	45.00	45.00	45.00	45.00
**A22 IRQ-21**	8094	45.00	45.00	45.00	30.77	45.00	45.00	45.00	29.03	45.00	45.00	45.00	45.00	45.00	45.00	45.00	24.61	X	X	X	45.00	45.00	45.00	45.00	27.03
	8095	45.00	32.75	45.00	31.00	45.00	45.00	45.00	31.75	45.00	45.00	45.00	45.00	45.00	45.00	45.00	30.30	X	X	X	32.69	45.00	45.00	45.00	30.69
	8096	45.00	45.00	45.00	29.71	45.00	45.00	45.00	29.91	45.00	45.00	45.00	45.00	45.00	45.00	45.00	28.68	X	X	X	30.27	45.00	45.00	45.00	31.54
	8097	45.00	30.03	45.00	30.08	45.00	45.00	45.00	28.19	45.00	45.00	45.00	45.00	45.00	45.00	45.00	27.42	X	X	X	29.85	45.00	45.00	45.00	26.34
	8098	45.00	45.00	45.00	31.17	45.00	45.00	45.00	45.00	45.00	45.00	45.00	30.58	45.00	45.00	45.00	28.08	X	X	X	27.62	45.00	45.00	45.00	28.95
**A MAY97-21**	8099	45.00	45.00	45.00	30.45	45.00	45.00	45.00	30.17	45.00	45.00	45.00	25.65	45.00	45.00	45.00	28.56	X	X	X	30.90	45.00	45.00	45.00	31.06
	8100	45.00	45.00	45.00	29.42	45.00	45.00	45.00	31.83	45.00	45.00	45.00	28.29	45.00	45.00	45.00	26.73	X	X	X	26.38	45.00	45.00	45.00	28.07
	8101	45.00	30.29	45.00	28.90	45.00	45.00	45.00	31.06	45.00	45.00	45.00	30.87	45.00	45.00	45.00	28.74	X	X	X	27.56	45.00	45.00	45.00	45.00
	8102	45.00	33.17	45.00	45.00	45.00	45.00	31.86	28.48	45.00	45.00	45.00	45.00	45.00	45.00	45.00	26.88	X	X	X	45.00	45.00	45.00	45.00	29.30
	8103	45.00	33.17	45.00	29.88	45.00	45.00	32.05	27.47	45.00	45.00	45.00	29.63	45.00	45.00	45.00	45.00	X	X	X	X	X	X	X	X
**A22 IRQ-7**	8104	45.00	33.08	45.00	45.00	45.00	45.00	45.00	26.53	45.00	45.00	45.00	45.00	45.00	45.00	45.00	27.73	X	X	X	25.81	45.00	45.00	45.00	30.17
	8105	45.00	45.00	45.00	31.17	45.00	45.00	45.00	30.95	45.00	45.00	45.00	30.08	45.00	45.00	45.00	30.61	X	X	X	31.17	45.00	45.00	45.00	31.82
	8106	45.00	30.56	45.00	28.75	45.00	32.45	45.00	27.05	45.00	45.00	45.00	29.42	45.00	45.00	45.00	22.93	X	X	X	23.16	45.00	45.00	45.00	28.02
	8107	45.00	45.00	45.00	31.98	45.00	45.00	45.00	45.00	45.00	45.00	45.00	31.34	45.00	45.00	45.00	29.01	X	X	X	29.70	45.00	45.00	45.00	29.43
	8108	45.00	45.00	45.00	45.00	45.00	45.00	45.00	24.46	45.00	45.00	45.00	45.00	45.00	45.00	45.00	25.62	X	X	X	27.56	45.00	45.00	45.00	28.79
**A MAY97-7**	8109	45.00	32.91	45.00	28.48	45.00	31.90	45.00	30.55	45.00	45.00	45.00	27.73	45.00	33.01	45.00	28.00	X	X	X	25.17	45.00	45.00	45.00	26.05
	8110	45.00	32.96	45.00	45.00	45.00	45.00	45.00	27.97	45.00	45.00	45.00	45.00	45.00	45.00	45.00	29.87	X	X	X	45.00	45.00	45.00	45.00	28.08
	8111	45.00	29.69	45.00	26.98	45.00	32.86	45.00	26.13	45.00	45.00	45.00	28.82	45.00	45.00	45.00	24.46	X	X	X	26.20	45.00	45.00	45.00	31.46
	8112	45.00	33.11	45.00	29.85	45.00	45.00	45.00	30.01	45.00	45.00	45.00	25.57	45.00	45.00	45.00	23.59	X	X	X	26.66	45.00	45.00	45.00	23.87
**UVC**	8113	45.00	31.63	45.00	30.46	45.00	45.00	45.00	30.85	45.00	45.00	45.00	28.65	45.00	45.00	45.00	27.94	X	X	X	29.47	45.00	45.00	45.00	29.15
	8114	45.00	26.83	32.45	23.90	Animal Euthanised																			

dpc – day post challenge; S – Serum, OS – Oral Swab, NS – Nasal Swab, P – Probang sample,
Cells with grey shade are positive for FMDV genome; Numerical values indicate the Cq values of qPCR; X – No sampling done on that day and * - Cattle that were not protected upon challenge

Supplementary data 1: Probability of protection

Day 21 groups

	Protected	Not protected
A22 IRQ-21	5	0
AMAY97-21	5	0

Fisher's Exact Test for Count Data p-value = 1

Day 7 groups

	Protected	Not protected
A22 IRQ-7	4	1
AMAY97-7	3	2

Fisher's Exact Test for Count Data p-value = 1

Supplementary data 2: Analysis of complete data set A-SEA97 variant challenge studies using A22 IRQ and A MAY97 vaccines

VNT data:

A22 IRQ serology between groups 21 day and 7 day

Two Sample t-test

data: data[group == 1 | group == 3,]$AbA22 by factor(data[group == 1 | group == 3,]$group)

t = 4.4956, df = 8, p-value = 0.002014

alternative hypothesis: true difference in means is not equal to 0

95 percent confidence interval: 0.3506792, 1.0893208

sample estimates:

mean in group 1	mean in group 3
2.22	1.50

A MAY97 serology between groups 21 day and 7 day

Two Sample t-test

data: data[group == 2 | group == 4,]$AbAMAY by factor(data[group == 2 | group == 4,]$group)

t = 3.0921, df = 8, p-value = 0.01484

alternative hypothesis: true difference in means is not equal to 0

95 percent confidence interval: 0.1037737, 0.7332263

sample estimates:

mean in group 2	mean in group 4
2.01	1.59

A VIT15 serology between A22 IRQ groups

Two Sample t-test

data: data[group == 1 | group == 3,]$AbVIT by factor(data[group == 1 | group == 3,]$group)

t = 2, df = 8, p-value = 0.08052

alternative hypothesis: true difference in means is not equal to 0

95 percent confidence interval: -0.04590062, 0.64590062

sample estimates:

mean in group 1	mean in group 3
1.35	1.05

A VIT15 serology between A MAY97 groups

Two Sample t-test

data: data[group == 2 | group == 4,]$AbVIT by factor(data[group == 2 | group == 4,]$group)

t = 2.1999, df = 8, p-value = 0.059

alternative hypothesis: true difference in means is not equal to 0

95 percent confidence interval: -0.02025961, 0.86025961

sample estimates:

mean in group 2	mean in group 4
1.11	0.69

A VIT15 serology: Comparison of vaccine groups both vaccinated 21 days before challenge

Two Sample t-test

data: data[group == 1 | group == 2,]$AbVIT by factor(data[group == 1 | group == 2,]$group)

t = 1.4254, df = 8, p-value = 0.1919

alternative hypothesis: true difference in means is not equal to 0

95 percent confidence interval: -0.1482725, 0.6282725

sample estimates:
mean in group 1 mean in group 2
1.35 1.11

A VIT15 serology: Comparison of vaccine groups both vaccinated 7 days before challenge

Two Sample t-test
data: data[group == 3 | group == 4,]$AbVIT by factor(data[group == 3 | group == 4,]$group)
t = 2.058, df = 8, p-value = 0.07359
alternative hypothesis: true difference in means is not equal to 0
95 percent confidence interval: -0.04338598, 0.76338598

sample estimates:
mean in group 3 mean in group 4
1.05 0.69

Figure 2: ANOVA results that were significant were analysed by pairwise comparison

Comparison of A22 Antibody response in A22 vaccinated cattle and control cattle (group 1, 3 and 5)
summary(aov(data[group!=2&group!=4,]$AbA22~data[group!=2&group!=4,]$group))

	df	Sum Sq	Mean Sq	F value	Pr(>F)
data[group != 2 & group != 4,]$group	2	8.044	4.022	78.4	7.74e-07 ***
Residuals	10	0.513	0.051		

Signif. codes: 0 ‘***’ 0.001 ‘**’ 0.01 ‘*’ 0.05 ‘.’ 0.1 ‘ ’ 1

Pairwise comparisons using t tests with non-pooled SD
data: data[group != 2 & group != 4,]$AbA22 and data[group != 2 & group != 4,]$group

	Group 1	Group 3
Group 3	0.0029	-
Group 5	5.7e-05	0.0011

P value adjustment method: holm

Comparison of AMAY Antibody response in AMAY vaccinated cattle and control cattle (group 2, 4 and 5)
Pairwise comparisons using t tests with non-pooled SD
data: data[group != 1 & group != 3,]$AbAMAY and data[group != 1 & group != 3,]$group

	Group 2	Group 4
Group 4	0.01511	-
Group 5	9.7e-05	0.00029

P value adjustment method: holm

Comparison of AVIT Antibody response in -21 days vaccinated cattle and control cattle (group 1, 2 and 5)

summary(aov(data[group!=3&group!=4,]$AbVIT~data[group!=3&group!=4,]$group))

	df	Sum Sq	Mean Sq	F value	Pr(>F)
data[group != 3 & group != 4,]$group	2	2.836	1.4178	25.01	0.000128 ***
Residuals	10	0.567	0.0567		

Signif. codes: 0 ‘***’ 0.001 ‘**’ 0.01 ‘*’ 0.05 ‘.’ 0.1 ‘ ’ 1

Pairwise comparisons using t tests with non-pooled SD
data: data[group != 3 & group != 4,]$AbVIT and data[group != 3 & group != 4,]$group

	Group 1	Group 2
Group 2	0.1923	-
Group 5	0.0020	0.0021

P value adjustment method: holm

Comparison of AVIT Antibody response in -7 days vaccinated cattle and control cattle (group 3, 4 and 5)

summary(aov(data[group!=1&group!=2,]$AbVIT~data[group!=1&group!=2,]$group))

	df	Sum Sq	Mean Sq	F value	Pr(>F)
data[group != 1 & group != 2,]$group	2	1.520	0.7602	12.42	0.00195 **

Residuals 10 0.612 0.0612

Signif. codes: 0 ‘***’ 0.001 ‘**’ 0.01 ‘*’ 0.05 ‘.’ 0.1 ‘ ’ 1

Pairwise comparisons using t tests with non-pooled SD

data: data[group != 1 & group != 2,]$AbVIT and data[group != 1 & group != 2,]$group

	Group 3	Group 4
Group 4	0.0846	-
Group 5	0.0012	0.0499

P value adjustment method: holm

Supplementary data 3: Linear Mixed Models

NSP Data:

The best fitting linear mixed effects model was select by forward selection.
The best fitting model was: Linear mixed model fit by REML ['lmerMod']
Formula: nsp ~ factor(dpc) + group + group:factor(dpc) + (1 | Animal)

REML criterion at convergence: 716.2

Scaled residuals:

Min	1Q	Median	3Q	Max
-3.3626	-0.3321	0.0117	0.4522	3.3626

Random effects:

Groups Name	Variance	Std.Dev.
Animal (Intercept)	19.52	4.419
Residual	30.84	5.553

Number of obs: 133, groups: Animal, 23

Fixed effects:

	Estimate	Std. Error	t value
(Intercept)	72.000	3.174	22.686
factor(dpc)10	11.200	3.512	3.189
factor(dpc)14	12.200	3.512	3.473
factor(dpc)21	8.600	3.512	2.449
factor(dpc)28	12.000	3.512	3.417
factor(dpc)35	12.400	3.512	3.530
group 2	-11.400	4.488	-2.540
group 3	-0.400	4.488	-0.089
group 4	-8.400	4.488	-1.871
group 5	-3.000	5.183	-0.579
factor(dpc)10:group 2	7.000	4.967	1.409
factor(dpc)14:group 2	6.800	4.967	1.369
factor(dpc)21:group 2	12.200	4.967	2.456
factor(dpc)28:group 2	11.600	4.967	2.335
factor(dpc)35:group 2	11.200	4.967	2.255
factor(dpc)10:group 3	3.400	4.967	0.684
factor(dpc)14:group 3	4.400	4.967	0.886
factor(dpc)21:group 3	6.200	4.967	1.248
factor(dpc)28:group 3	1.800	4.967	0.362
factor(dpc)35:group 3	2.434	5.143	0.473
factor(dpc)10:group 4	3.200	4.967	0.644
factor(dpc)14:group 4	5.600	4.967	1.127
factor(dpc)21:group 4	10.400	4.967	2.094
factor(dpc)28:group 4	9.400	4.967	1.892
factor(dpc)35:group 4	10.000	4.967	2.013
factor(dpc)10:group 5	7.133	5.736	1.244
factor(dpc)14:group 5	9.114	6.283	1.451
factor(dpc)21:group 5	10.214	6.283	1.626
factor(dpc)28:group 5	-15.186	6.283	-2.417
factor(dpc)35:group 5	-21.086	6.283	-3.356

Correlation matrix not shown by default, as p = 30 > 12.

VI and PCR data
(Linear mixed effects models were select by forward selection)

Comparison of VI data from blood

```
m0<-lmer(VI~1+(1|animal),data=dat[sample=='blood',])
m1<-lmer(VI~group+(1|animal),data=dat[sample=='blood',])
m2<-lmer(VI~DPC+(1|animal),data=dat[sample=='blood',])
AIC(m0,m1,m2)
```

	df	AIC
m0	3	15.51916
m1	4	17.44939
m2	5	17.92069

Comparison of RT-PCR data from blood

```
> m0<-lmer(PCR~1+(1|animal),data=dat[sample=='blood',])
> m1<-lmer(PCR~group+(1|animal),data=dat[sample=='blood',])
> m2<-lmer(PCR~DPC+(1|animal),data=dat[sample=='blood',])
> AIC(m0,m1,m2)
```

	df	AIC
m0	3	155.4926
m1	5	149.9067
m2	7	125.8333

```
> m3<-lmer(PCR~group+DPC+(1|animal),data=dat[sample=='blood',])
> AIC(m2,m3)
```

	df	AIC
m2	7	125.8333
m3	9	114.9344

```
> summary(m3)
```

Linear mixed model fit by REML ['lmerMod']
Formula: PCR ~ group + DPC + (1 | animal)
Data: dat[sample == "blood",]

REML criterion at convergence: 96.9
Scaled residuals:

Min	1Q	Median	3Q	Max
-2.1605	-0.4118	0.1501	0.5877	0.9923

Random effects:

Groups Name	Variance	Std.Dev.
animal (Intercept)	5.606	2.368
Residual	3.600	1.897

Number of obs: 26, groups: animal, 8

Fixed effects:

	Estimate	Std. Error	t value
(Intercept)	21.7062	1.6243	13.363
Group 1	7.4080	2.4953	2.969
Group 4	5.6515	2.1856	2.586
DPC 2	6.1415	1.2557	4.891
DPC 3	-0.3947	1.0731	-0.368
DPC 4	7.2717	1.1520	6.312
DPC 5	10.6238	1.4034	7.570

Correlation of Fixed Effects:

	(Intr)	group 1	group 4	DPC 2	DPC 3	DPC 4
Group 1	-0.595					
Group 4	-0.665	0.417				
DPC 2	-0.337	0.148	0.119			
DPC 3	-0.325	0.090	0.065	0.417		
DPC 4	-0.333	0.092	0.115	0.424	0.449	
DPC 5	-0.338	0.155	0.160	0.390	0.376	0.386

Comparison of VI data from oral swabs

```
> m0<-lmer(VI~1+(1|animal),data=dat[sample=='Mouth swabs',])
> m1<-lmer(VI~group+(1|animal),data=dat[sample=='Mouth swabs',])
> m2<-lmer(VI~DPC+(1|animal),data=dat[sample=='Mouth swabs',])
> AIC(m0,m1,m2)
```

	df	AIC
m0	3	546.4407
m1	7	552.5744
m2	9	471.3832

```
> m3<-lmer(VI~group+DPC+(1|animal),data=dat[sample=='Mouth swabs',])
> AIC(m2,m3)
```

	df	AIC
m2	9	471.3832
m3	13	475.4256

```
> summary(m2)
```

Linear mixed model fit by REML ['lmerMod']
Formula: VI ~ DPC + (1 | animal)
Data: dat[sample == "Mouth swabs",]

REML criterion at convergence: 453.4

Scaled residuals:

Min	1Q	Median	3Q	Max
-3.4795	-0.5686	-0.0351	0.5648	2.1715

Random effects:

Groups Name	Variance	Std.Dev.
animal (Intercept)	0.2837	0.5326
Residual	1.1301	1.0630

Number of obs: 146, groups: animal, 23

Fixed effects:

	Estimate	Std. Error	t value
(Intercept)	4.9013	0.2479	19.769
DPC2	-0.2683	0.3135	-0.856
DPC3	-1.1830	0.3135	-3.774
DPC4	-1.3017	0.3135	-4.153
DPC5	-1.7304	0.3135	-5.520
DPC6	-2.7572	0.3263	-8.451
DPC7	-3.3447	0.3966	-8.433

Correlation of Fixed Effects:

	(Intr)	DPC2	DPC3	DPC4	DPC5	DPC6
DPC2	-0.632					
DPC3	-0.632	0.500				
DPC4	-0.632	0.500	0.500			
DPC5	-0.632	0.500	0.500	0.500		
DPC6	-0.607	0.480	0.480	0.480	0.480	
DPC7	-0.500	0.395	0.395	0.395	0.395	0.386

Comparison of RT-PCR data from oral swabs

```
> m0<-lmer(PCR~1+(1|animal),data=dat[sample=='Mouth swabs',])
> m1<-lmer(PCR~group+(1|animal),data=dat[sample=='Mouth swabs',])
> m2<-lmer(PCR~DPC+(1|animal),data=dat[sample=='Mouth swabs',])
> AIC(m0,m1,m2)
```

	df	AIC
m0	3	1181.836
m1	7	1177.021
m2	12	1003.513

```
> m3<-lmer(PCR~group+DPC+(1|animal),data=dat[sample=='Mouth swabs',])
> AIC(m2,m3)
```

	df	AIC
m2	12	1003.5125
m3	16	996.4007

```
> summary(m3)
```

Linear mixed model fit by REML ['lmerMod']
Formula: PCR ~ group + DPC + (1 | animal)
Data: dat[sample == "Mouth swabs",]

REML criterion at convergence: 964.4

Scaled residuals:

Min	1Q	Median	3Q	Max
-2.5439	-0.5796	-0.0820	0.5263	3.2251

Random effects:

Groups Name	Variance	Std.Dev.
animal (Intercept)	4.58	2.140
Residual	16.28	4.035

Number of obs: 175, groups: animal, 23

Fixed effects:

	Estimate	Std. Error	t value
(Intercept)	12.4892	1.6910	7.386
group1	2.1410	1.8982	1.128
group2	3.3093	1.9014	1.740
group3	0.4342	1.8917	0.230
group4	1.8230	1.8962	0.961
DPC2	0.7609	1.1898	0.640
DPC3	13.1919	1.2380	10.656
DPC4	6.0117	1.1898	5.053
DPC5	6.3836	1.2047	5.299
DPC6	8.8161	1.1898	7.410
DPC7	10.3943	1.1898	8.736
DPC10	18.0155	1.3852	13.006
DPC14	18.9131	2.5673	7.367
DPC28	19.5928	4.2846	4.573

Correlation matrix not shown by default, as p = 14 > 12.

Comparison of VI data from nose swabs

```
> m0<-lmer(VI~1+(1|animal),data=dat[sample=='Nose swabs',])
> m1<-lmer(VI~group+(1|animal),data=dat[sample=='Nose swabs',])
> m2<-lmer(VI~DPC+(1|animal),data=dat[sample=='Nose swabs',])
> AIC(m0,m1,m2)
```

	df	AIC
m0	3	114.7409
m1	7	114.3589
m2	8	114.5556

Comparison of RT-PCR data from nose swabs

```
> m0<-lmer(PCR~1+(1|animal),data=dat[sample=='Nose swabs',])
> m1<-lmer(PCR~group+(1|animal),data=dat[sample=='Nose swabs',])
> m2<-lmer(PCR~DPC+(1|animal),data=dat[sample=='Nose swabs',])
> AIC(m0,m1,m2)
```

	df	AIC
m0	3	800.9816
m1	7	796.6992
m2	11	551.2070

```
> m3<-lmer(PCR~group+DPC+(1|animal),data=dat[sample=='Nose swabs',])
> AIC(m2,m3)
```

	df	AIC
m2	11	551.2070
m3	15	545.0977

```
> summary(m3)
Linear mixed model fit by REML ['lmerMod']
Formula: PCR ~ group + DPC + (1 | animal)
Data: dat[sample == "Nose swabs", ]
```

REML criterion at convergence: 515.1

Scaled residuals:

Min	1Q	Median	3Q	Max
-3.4429	-0.3864	0.1062	0.5082	1.9993

Random effects:

Groups Name	Variance	Std.Dev.
animal (Intercept)	1.903	1.380
Residual	2.880	1.697

Number of obs: 130, groups: animal, 23

Fixed effects:

	Estimate	Std. Error	t value
(Intercept)	27.92455	0.98405	28.377
Group 1	2.56794	1.12428	2.284
Group 2	2.78541	1.13831	2.447
Group 3	2.66716	1.12582	2.369
Group 4	1.74425	1.12733	1.547
DPC 2	-2.01564	0.61064	-3.301
DPC 3	-11.60341	0.60311	-19.239
DPC 4	0.05903	0.61019	0.097
DPC 5	0.98235	0.63327	1.551
DPC 6	1.65799	0.64643	2.565
DPC 7	1.87082	0.67959	2.753
DPC 10	6.21257	1.85358	3.352
DPC 14	1.60302	1.38754	1.155

Correlation matrix not shown by default, as p = 13 > 12.

Comparison of VI data from probang samples

```
> tab    ##VI probang number of animals with a positive result
```

	positive	negative
A22-21	2	3
AMAY-21	4	1
A22-7	4	1
AMAY-7	3	2
controls	2	1

```
> fisher.test(tab)
Fisher's Exact Test for Count Data
data:  tab
p-value = 0.8572
alternative hypothesis: two.sided

> m0<-lmer(VI~1+(1|animal),data=dat[sample=='probang',])
> m1<-lmer(VI~group+(1|animal),data=dat[sample=='probang',])
> m2<-lmer(VI~DPC+(1|animal),data=dat[sample=='probang',])
> AIC(m0,m1,m2)
     df  AIC
m0   3   39.50920
m1   7   44.41957
m2   8   48.14905
```

Comparison of RT-PCR data from probang samples

```
> m0<-lmer(PCR~1+(1|animal),data=dat[sample=='probang',])
> m1<-lmer(PCR~group+(1|animal),data=dat[sample=='probang',])
> m2<-lmer(PCR~DPC+(1|animal),data=dat[sample=='probang',])
> AIC(m0,m1,m2)
     df  AIC
m0   3   673.3149
m1   7   665.7474
m2   9   632.3202

> m3<-lmer(PCR~group+DPC+(1|animal),data=dat[sample=='probang',])
> AIC(m2,m3)
     df  AIC
m2   9   632.3202
m3   13  624.0570

> summary(m3)
Linear mixed model fit by REML ['lmerMod']
Formula: PCR ~ group + DPC + (1 | animal)
Data: dat[sample == "probang", ]

REML criterion at convergence: 598.1

Scaled residuals:
Min       1Q        Median    3Q       Max
-2.33736  -0.54251  0.04665   0.58324  2.39152

Random effects:
Groups Name          Variance  Std.Dev.
animal (Intercept)   1.40      1.183
Residual             5.31      2.304
Number of obs: 133, groups:  animal, 23
Fixed effects:
             Estimate  Std. Error  t value
(Intercept)  22.9302   1.0136      22.622
Group 1      3.4836    1.1492      3.031
Group 2      2.6230    1.1411      2.299
Group 3      1.8449    1.1587      1.592
Group 4      1.8275    1.1523      1.586
DPC 10       4.5661    0.7194      6.347
DPC 14       3.8867    0.7190      5.406
DPC 21       4.0892    0.7927      5.159
DPC 28       2.2787    0.6984      3.263
DPC 30       3.1980    0.7316      4.372
DPC 35       3.6712    0.7192      5.105
```

Correlation of Fixed Effects:

	(Intr)	Group 1	Group 2	Group 3	Group 4	DPC 10	DPC 14	DPC 21	DPC 28	DPC 30
Group 1	-0.726									
Group 2	-0.727	0.652								
Group 3	-0.724	0.642	0.647							
Group 4	-0.726	0.647	0.651	0.643						
DPC 10	-0.334	0.003	0.004	0.031	0.031					
DPC 14	-0.292	-0.021	-0.022	-0.035	-0.023	0.442				
DPC 21	-0.283	0.008	-0.021	-0.004	-0.002	0.415	0.404			
DPC 28	-0.297	-0.033	-0.032	-0.019	-0.033	0.458	0.464	0.419		
DPC 30	-0.293	-0.020	-0.033	-0.004	-0.019	0.443	0.440	0.409	0.460	
DPC 35	-0.293	-0.021	-0.022	-0.020	-0.034	0.442	0.453	0.403	0.467	0.445
